# Balaraj Maharishi and the first clinical trial of Ayurvedic medicines in the West

**DOI:** 10.4103/0975-9476.72615

**Published:** 2010

**Authors:** Donn Brennan

**Affiliations:** *Medical Doctor and Ayurveda Practitioner, Dublin, UK*

In June 1984, I was part of a group of western-trained medical doctors from six countries who began a 15-month course in Ayurveda. In February 1985 as part of our course, we were invited to join a group of *Vaidyas* in Brasilia, Brazil, for a two-week conference on the indigenous health traditions of South America. It was here that I first came into contact with Balaraj Maharishi, one of the great Vaidyas of his era, and at that time adviser on Ayurveda to the Government of Andhra Pradesh.

In Brasilia, he soon came to the notice of our group, but in an unusual way. Conference sessions would last many hours with the Chairman, Maharishi Mahesh Yogi, and others speaking. As we sat watching the proceedings, we could not help in noticing that one person on the stage sat for hours hardly moving, or without moving at all. The stillness surrounding his presence was palpable. Even after the first session, our group all wanted to know who he might be.

We were told that this was Balaraj Maharishi, a senior and highly respected Vaidya, a great living authority on Ayurvedic medicinal plants and their uses – the science of *Dravyaguna*. At the meeting that was arranged, Balaraj Maharishi told us something of his life story. As a 17-year-old, he had been travelling by train in North India when he witnessed a train guard demanding payment for his fare from a *Sannyasi*, something that never usually happened. He remonstrated with the guard, but ended by paying the man’s fare for him. This had much amused the *Sannyasi*, who asked the young man what he intended to do with his life. Balaraj said he just wished to make people happy and so was considering music. He had run away from home and was on his way to Madras to learn a traditional instrument from a group who had recently visited his village.

On hearing this, the *Sannyasi* offered to teach him something more precious, and invited Balaraj to follow him. It turned out that he was an experienced Ayurvedic doctor with life-time knowledge of medicinal plants and their uses. In this way, as a teenager, Balaraj began to learn Ayurveda from a *Vaidya Sannyasi*, who had invited him to become his *shishya* (student) at their very first meeting.

From then on, wherever they walked through forests, fields, or deserts of India, but particularly in the Himalayas, every time they met a plant his master would tell him all about it – names, family, genus, properties, uses, in what combinations it could be used, and for what conditions, etc. For many years, they walked the length and breadth of India, particularly the Himalayas, with his instruction continuing. He had thus acquired detailed working knowledge of some 4000 plants, or so it was reputed.

One day in Brasilia, it was decided that the visiting *Vaidyas* would join a group of traditional practitioners from South America on a field trip into the jungle to study local plants. By the end of the day, Balaraj Maharishi had earned the respect of all. Whenever they had come to a plant whose identity or health benefits were unknown to all others, Balaraj would explain everything about it, Sanskrit name, Latin name, common name, and uses of its different parts. His knowledge seemed encyclopedic. He was subsequently described as “*sarvagyan oushadhi”* – having universal knowledge of plants.

Thirty years after their first meeting, his teacher told him to go and give his knowledge to the world. His own professional career had thus started after many years training with his *Sannyasi* guru. Balaraj ran camps in rural India, and his reputation began to grow. His *modus operandi* was to hold a camp for a week, during which many thousands of sick people would come. He and his growing number of *Vaidya* apprentices would work day and night treating them the entire week. He would treat the more difficult patients himself. After the week’s camp, the following three weeks would be spent collecting plants and processing them for the next camp.

Balaraj’s closest student in his healing work was Dr. Raju from Hyderabad, whom we also met in Brasilia. Raju explained how the tribal peoples of the forests revered Balaraj – they would collect plants each month for him, and he, in return, would then visit them to treat and heal their ailments. He told us many stories of times when they were in the forest. For example, one rare plant called *bhutumbi*[1] is of great medicinal value, but cobras like to build their nests around its roots to such an extent that cobras are usually found wherever it grows. Those trying to harvest *bhutumbi* are in danger of snakebite from the disturbed snakes who naturally do not wish to see their home destroyed. So how to pick it? The answer was stunningly simple, literally! Balaraj would throw a powder into the nest, which would stun all the snakes for half an hour. Then they would dig out the plant and Balaraj would throw another powder on the snakes. I asked why the second powder? Raju said, in case any snake was cut in the process of digging, the second powder would help to heal its wounds!

Balaraj ended his life’s story by saying that no year went by without his returning to visit his guru in the Himalayas. When asked if he meant the old Sannyasi he had met him at the age of 17, he consistently said, “yes, of course.” In 1985, Balaraj was said to be in his seventies, so it must have been about 60 years later. Raju also confirmed that Balaraj used to visit his guru annually up to a few years before he passed away in 2001. The age of his revered guru must have been beyond all normal life-expectations.

On other occasions, Balaraj Maharishi revealed further aspects of his skills and his life. On one occasion in India, he was shown a sprig of oleander which had been growing in the courtyard of the place where he was staying. Far from being just a beautiful ornamental, oleander is exceedingly toxic. In addition to the well-known oleandrin, atropine, and strychnine, it contains dozens of other cardioglycosides, all of which have been found to be fatal. So much so that western medicine recognizes no beneficial dosage level for its extracts. When asked whether he knew any use for it, Balaraj held the sprig in his hand and considered it carefully. “Yes,” he replied, “if you soak the leaves in lemon juice overnight, and then boil the solution until it forms a jelly on cooling, a pin head of that jelly will be a cure for malaria.”

Balaraj had had a very high reputation for many years. He once related how, after independence in 1947, when the British were finally leaving India, Lord Mountbatten, the last Viceroy, had requested him to accompany their group back to England, saying that they needed people of his quality. He had declined on the grounds that his duty lay with his own people in his homeland. On another occasion, he opined to one of my colleagues that people in the west “should now receive the full wisdom of Ayurveda.” This project was one to which he later gave considerable support, conducting a successful clinical trial of Ayurvedic medicines in the west.[2]

To help spread the knowledge of Ayurveda internationally, Balaraj travelled widely to Europe and the Americas, instructing qualified doctors in the use of Ayurvedic herbs. One day, somebody asked him a question, “Do you know a single formula which is good for every disease?” Balaraj replied that just such a question had once been posed in ancient times by a great King to his entire team of *Rajavaidyas*. Their answer, which had never been written down, had been known to the *Sadhu Vaidya*, who had passed it to him many years earlier, with instructions that it should be used to benefit all humanity. Balaraj Maharishi first named the product “Amrit Kalash,” since he first presented it in a silver *kalash* container, describing it as pure *Amrit* (nectar). Thus came about the Maharishi *Amrit Kalash Ambrosia* Tablets.

From the perspective of modern science and integrative medicine, Balaraj’s greatest contribution may well have been his supervision of one of the first published formal scientific trials of Ayurvedic treatments conducted in a foreign country.[2] To validate the efficacy of Balaraj’s herbal formulae, a trial of chronic diseases was set up by a Dutch scientist, the late George Janssen, in Holland. The trial was conducted under the supervision of a Dutch health insurance company, Zilveren Kruis (Silver Cross). It monitored the effects of the herbal formulae on chronically ill patients with no hope of recovery. Since the patients were regarded as incurable in western medicine, and were long-term users of palliative drugs, any improvements in their underlying condition could only be due to the Ayurvedic treatment. For this reason, no controls were deemed necessary. Despite such negative circumstances, the results of Balaraj Maharishi’s individualized Ayurvedic treatments were very successful.[2] Zilveren Kruis stated that they would be willing to pay for any of their insurees suffering from the pathologies investigated to be treated with Balaraj’s Ayurvedic prescriptions.

Naturally, the patients had been given full Ayurvedic treatments, including *ahara/vihara* (diet/lifestyle) recommendations as well as *aushadi* (herbal medicines). This trial, therefore, does not in any way constitute a test of herbal formulae versus chemical drugs. It is a comparison of the full treatments of two potentially complementary medical systems in cases where one, modern medicine, is not able to improve the underlying condition. The conclusion that follows is that these constitute conditions where the integrative practice of the two systems of medicine together will greatly benefit overall treatment outcomes, and future quality of patient life. Balaraj’s research thus points to the advantages of adopting integrative practice so that more than one system is available to fit patient’s individual needs.

Balaraj had excellent rapport with his patients. One of those helping with the Zilveren Kruis study, Wim van den Berg, recalls his father, who did not speak any other language than Dutch, and was then about 80 years old, coming to consult as part of the study. Balraj, he recalls, sat in his office like a King, receiving patient after patient with the aura of an elderly and wise saint, helped by a translator.

Wim van den Berg recalls an amazing intuitive contact and understanding between the two men, almost without any verbal interaction. Although he translated a few things here and there, it was as if the two of them spoke from heart to heart at an almost silent level, as well as actively with gestures and sounds of all kinds. Balraj seemed to immediately understand the other man’s problems, and as if his father had just come to meet an old friend. His father had never previously expressed interest in Ayurveda, and Wim had been surprised at his willingness to participate in the study. Then, on meeting Balraj, his father had melted and become enthusiastic to take Ayurvedic medicine.
